# Shedding patterns of dairy calves experimentally infected with *Mycobacterium avium* subspecies *paratuberculosis*

**DOI:** 10.1186/s13567-014-0071-1

**Published:** 2014-08-08

**Authors:** Rienske AR Mortier, Herman W Barkema, Karin Orsel, Robert Wolf, Jeroen De Buck

**Affiliations:** 1 Department of Production Animal Health, University of Calgary, Hospital Drive NW, Calgary, AB, Canada

## Abstract

Although substantial fecal shedding is expected to start years after initial infection with *Mycobacterium avium* subspecies *paratuberculosis* (MAP), the potential for shedding by calves and therefore calf-to-calf transmission is underestimated in current Johne’s disease (JD) control programs. Shedding patterns were determined in this study in experimentally infected calves. Fifty calves were challenged at 2 weeks or at 3, 6, 9 or 12 months of age (6 calves served as a control group). In each age group, 5 calves were inoculated with a low and 5 with a high dose of MAP. Fecal culture was performed monthly until necropsy at 17 months of age. Overall, 61% of inoculated calves, representing all age and dose groups, shed MAP in their feces at least once during the follow-up period. Although most calves shed sporadically, 4 calves in the 2-week and 3-month high dose groups shed at every sampling. In general, shedding peaked 2 months after inoculation. Calves inoculated at 2 weeks or 3 months with a high dose of MAP shed more frequently than those inoculated with a low dose. Calves shedding frequently had more culture-positive tissue locations and more severe gross and histological lesions at necropsy. In conclusion, calves inoculated up to 1 year of age shed MAP in their feces shortly after inoculation. Consequently, there is potential for MAP transfer between calves (especially if they are group housed) and therefore, JD control programs should consider young calves as a source of infection.

## Introduction

Paratuberculosis or Johne’s disease (JD) is a chronic enteritis of ruminants caused by *Mycobacterium avium* subspecies *paratuberculosis* (MAP) [1]. The disease is widespread in dairy herds worldwide and causes substantial economic losses [2],[3], due to reduced milk yield [4],[5], premature culling and reduced slaughter value [6],[7]. If not culled before clinical signs appear after a long incubation period (years) [8], cattle suffer from chronic, non-treatable diarrhea which leads to cachexia and ultimately culling or death [1]. The primary route of MAP transmission is fecal-oral, usually through ingestion of water, milk, or feed, contaminated by ruminants shedding MAP in their feces [1].

Poor manure management, a contaminated environment for calves, and contact with a shedding dam are the main sources of MAP infection on a farm [9]–[11]. Therefore, JD control programs involve 2 main objectives: reduce the number of infected animals that are shedding MAP, and prevent fecal-oral transmission by implementing best-hygiene practices for newborn calves [7]. Although research studies have described associations between management practices and the probability of cattle being infected with MAP [12]–[14], specific questions remain unanswered. In particular, the potential of calves shedding and contaminating the environment, as well as the risk of calf-to-calf transmission is largely overlooked in the current JD prevention and control programs. Furthermore, only 1 of 8 MAP modeling studies included calf-to-calf transmission [15],[16]. Even though most reports only claim transmission between adults and calves [17], recent reports suggest calves can be infected by other calves [18],[19]. These contradicting results can be explained by the delayed onset of clinical disease and the low sensitivity of diagnostic tests in the early stages after MAP infection [8]. Van Roermund et al. reported that calf-to-calf transmission occurred, and that contact with infectious calves increased the possibility of other calves being MAP-infected [19]. However, it is not known how often and when these calves are shedding in relation to initial infection. Consequently, there is a need for longitudinal studies determining how often infected calves shed MAP.

The objective of the current study was to determine shedding patterns in calves inoculated with 2 doses of MAP at 5 ages. Impact of age and dose at time of inoculation on shedding patterns, as well as on interval to first positive fecal culture, were assessed. Finally, the frequency of fecal shedding was related to the severity of tissue lesions in the same calves.

## Materials and methods

### Herds and calves

Study design and sample collection were described in detail by Mortier et al. [20]. Briefly, male calves were collected from low MAP prevalence herds (<5% seropositive and < 5% fecal culture-positive) in Southern Alberta (Canada) and included in the study when born in the presence of a member of our research team. All dams were MAP ELISA (IDEXX Paratuberculosis Ab Test; IDEXX Laboratories Inc, Westbrook, ME, USA) and fecal culture-negative.

Upon arrival at the research facility, calves were fed 6 L of gamma-irradiated colostrum within 6 hours after birth. The colostrum used in this study was collected from ELISA-negative herds. This was followed by milk replacer and calf starter grain (without antimicrobial additives) and high-quality hay. Calves were individually housed under stringent biosecurity conditions and no contact was possible between calves. Calves were monitored until 17 months of age (+ or – 2 weeks). Consequently, calves inoculated at 2 weeks and 3, 6, 9, or 12 months were followed for 17, 14, 11, 8, and 5 months after inoculation, respectively. Health status was monitored on a daily basis by clinical inspection. At 17 months of age, euthanasia and necropsies were performed, including assessment for gross lesions, histology, and tissue culture [20]. Animal care protocols M09083 and M09050 were approved by the University of Calgary Animal Care Committee.

### Study design and inoculum

Study design and preparation of inoculum were described by Mortier et al. [20]. Fifty calves were randomly allocated to 5 age groups (2 weeks, 3, 6, 9 and 12 months). Six calves housed in the same conditions were not inoculated (negative controls). In each of the 5 age groups, 5 calves were inoculated per os with a high dose (HD) of MAP and 5 calves were inoculated with a low dose (LD) of MAP. Because of logistics, this experiment was performed in 2 replicates. The first and second replicates included 33 and 23 calves, respectively, with all age and dose groups represented in both replicates.

A virulent cattle type MAP strain isolated from a clinical Alberta JD case (Cow 69) was used for inoculation. This isolate had an identical BamHI, PvuII and PstI IS*900* – RFLP profile as the reference strain K10 recommended for use in infection trials [21]. Calves were challenged on 2 consecutive days, with either 5 × 10^9^ CFU (HD) or 5 × 10^7^ CFU (LD). The inoculum was prepared and cultured in 7H9 broth and quantified using the pelleted wet weight method. The quantification was confirmed using an in-house quantitative realtime PCR with a standard curve based on the 16*S* rRNA gene of *Mycobacterium smegmatis*, confirming the presence and the quantity of the 16*S* rRNA gene using primers p882 (5′-aggattagataccctggtag-3′) and p1100 (5′-gctgacgacatccatgc-3′).

### Fecal sampling and culture

Fecal samples were collected from the rectum from each calf prior to inoculation. Samples collected 1–5 days after inoculation were pooled (maximum of 3 samples per pool), containing samples from one calf collected over several days; this was an additional quality control measure to ensure viability of the inoculum, sensitivity of the fecal culture, and to confirm passive shedding of MAP [21],[22].

For the first 4 weeks after inoculation, rectal fecal samples were collected weekly; thereafter, fecal samples were collected monthly. To ensure age-matched control samples for each inoculation group, control calves were sampled twice per month.

All samples were processed using a modified TREK ESP II liquid culture system (TREK para-JEM®; TREK Diagnostic Systems, Cleveland, OH, USA) with subsequent IS*900* PCR on all samples. In more detail: From each fecal sample, 2 g was added to distilled water, mixed and allowed to settle for 30 min. Fecal samples were decontaminated according to manufacturer’s instructions. First, 5 mL of the settled mixture was added to 25 mL of a 0.9% hexadecylpyridinium chloride (HPC) in half strength brain heart infusion (BHI) solution and incubated overnight. Then, samples were centrifuged for 20 min at 3000 × *g*. The supernatant was discarded and the pellet was resuspended with a mixture of AS (ParaJem), water and full strength BHI. Tubes were incubated again for 24 h at 37 °C and added to the liquid culture medium (TREK para-JEM®; TREK Diagnostic Systems, Cleveland, OH, USA). After incubation for 48 days, MAP presence was confirmed by conventional PCR on culture medium targeting the IS*900* region. Extraction of DNA was done as described [23]. The IS*900* PCR procedure was modified from Vary et al. [24]; 5 μL of lysate was added to the described reaction mixture, containing 1.25 U Top Taq (Qiagen, Germantown, MD, USA), resulting in a reaction volume of 50 μL. Culture followed by PCR results were considered as a dichotomous outcome (MAP detected/not detected).

### Statistical analyses

All statistical analyses were performed using STATA 11 (StataCorpLP, College Station, TX, USA), with a *P*-value < 0.10 considered significant.

To distinguish between frequent shedding calves and sporadic shedding calves, categories were assigned based on the observed data: 0 = non-shedding calves, 1 = calves shedding 1–4 times during the follow-up, and 2 = calves shedding > 4 times during the follow-up period. Gross lesions, microscopic lesions and tissue culture results at necropsy were assigned to categories as described [20].

Differences in shedding between HD and LD calves, between age at inoculation, and among months after inoculation over time, as well as distributions of macroscopic and microscopic lesions and tissue culture results, between fecal shedding groups, were evaluated using Chi-square and Fisher’s Exact tests. Interval from inoculation to the first positive fecal culture was plotted using a Kaplan-Meier graph; groups were compared using the logrank test of equality [25].

## Results

### Sporadic and frequent shedding

**Figure 1:**
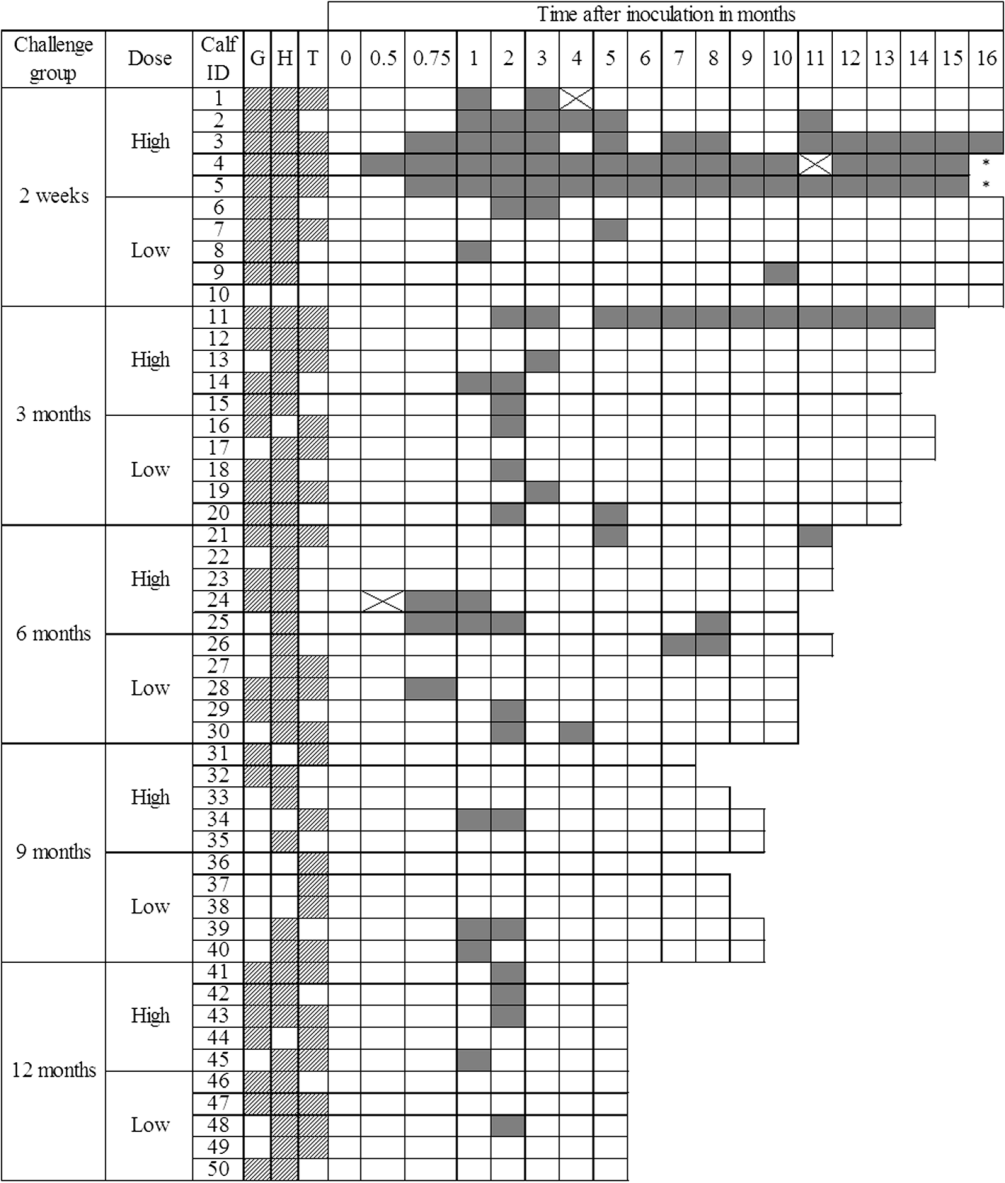
**Fecal culture results for individual calves per age and dose group.** A solid filled box indicates a positive fecal culture, a white box a negative culture and a box with a cross a missing sample. G = gross lesions, H = histology, and T = tissue culture at necropsy, boxes with shading indicate a positive sample. * = this calf developed clinical signs of JD.

All pre-inoculation fecal samples were MAP culture-negative. Passive shedding was detected 1–5 days after inoculation. No calf was fecal culture-positive at 7 days after inoculation and as of 2 weeks after inoculation, shedding was considered active. All calves tested negative at 2 weeks after inoculation, except for Calf 4 inoculated at 2 weeks of age with a HD, which started shedding 2 weeks after inoculation (Figure 1). Two calves (4 and 5; Figure 1) from the 2-week-HD group developed clinical JD; these calves consistently remained fecal culture-positive until they were euthanized (due to animal welfare concerns) at 16 months after inoculation (Figure 1).

In the control group that was followed until 17 months of age, 1 of 6 calves had 3 positive fecal cultures at 3.5, 4 and 7 months of age.

Shedding was detected at least once during the entire follow-up period in 32 (64%) of the 50 MAP-inoculated calves (Table 1, Figure 1) and occurred mostly sporadically in the shedding calves, with the exception of 4 calves that shed at most samplings Calves 3, 4, 5 and 11; Figure 1).

**Table 1:** Number and percentage of shedding calves in 5 age and 2 dose groups

	**2 weeks**	**3 months**	**6 months**	**9 months**	**12 months**	**Total**
Low dose	4* (80%)	4 (80%)	4 (80%)	2 (40%)	1(20%)	15 (60%)
High dose	5 (100%)	4 (80%)	3 (60%)	1 (20%)	4 (80%)	17 (68%)
Total	9 (90%)	8 (80%)	7 (70%)	3 (30%)	5 (50%)	

*5 calves total in each group; 50 calves in total.

### Impact of months after inoculation, dose, and age

**Figure 2:**
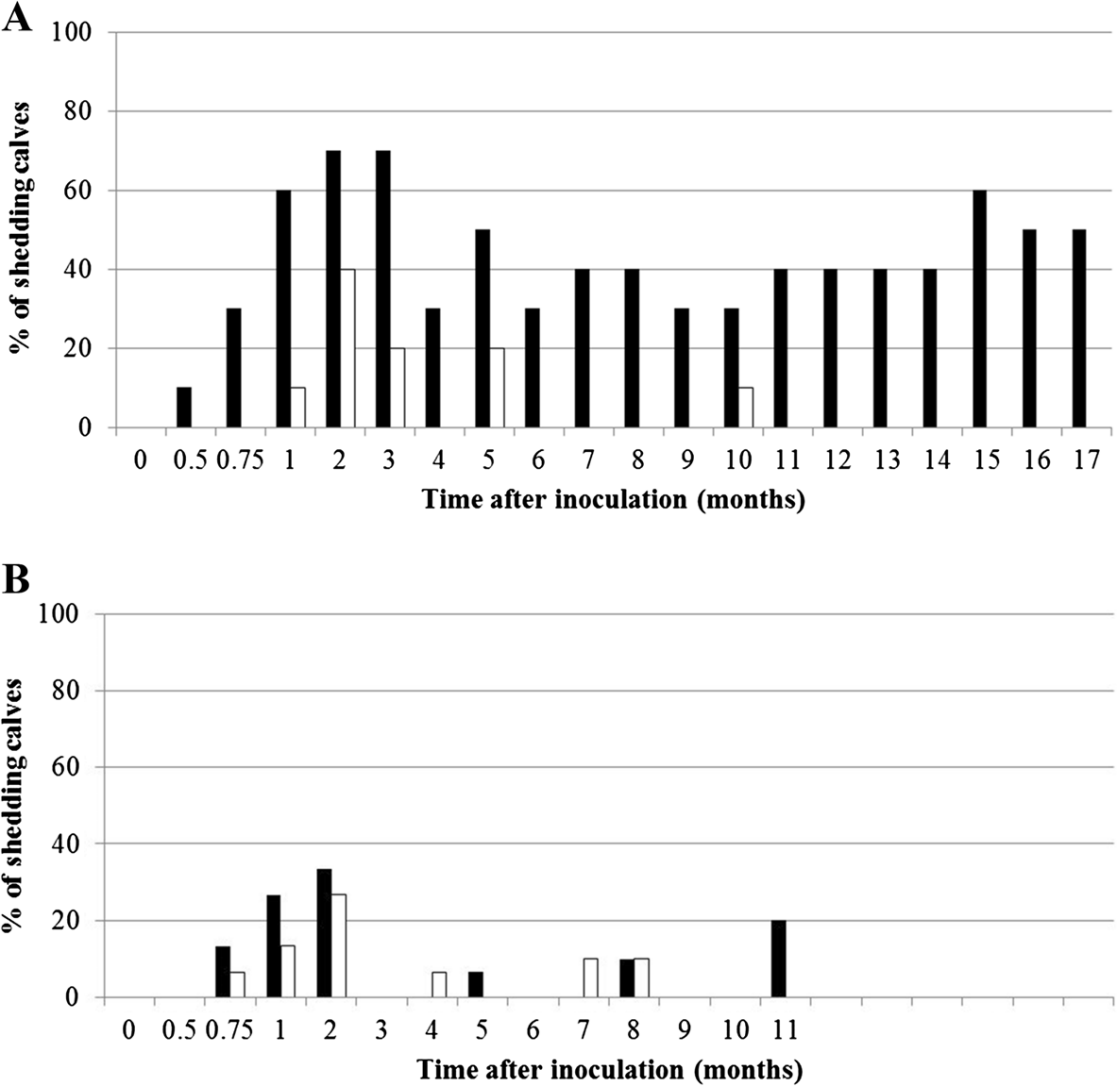
**Percentage fecal culture-positive calves in the 2 dose groups for every month after inoculation. A)** calves inoculated at 2 weeks or 3 months of age, and **B)** calves inoculated at 6, 9 or 12 months of age. Solid dark bars represent calves inoculated with a high dose of MAP and open white bars represent calves inoculated with a low dose of MAP. Note: the number of calves used to calculate this proportion decreases as the time after inoculation increases (calves inoculated at 2 weeks or 3, 6, 9 or 12 months were followed for 17, 14, 11, 8 and 5 months after inoculation, respectively).

Shedding peaked between inoculation and 6 months after inoculation, with the highest proportion (40%, 20 calves of 50) of calves shedding at 2 months after inoculation (*P* = 0.006; Figure 2).

Calves inoculated with a HD more frequently shed more than 4 times compared to calves inoculated with a LD (Figure 2) when inoculated at 2 weeks or 3 months of age (*P* = 0.04; Figure 2). Furthermore, all calves that shed more than 4 times were inoculated with a HD of MAP and at 2 weeks or 3 months of age. In groups inoculated at 6, 9, or 12 months of age, the proportion of calves that shed at least once was equal to non-shedding calves in those inoculated with either a HD or a LD of MAP (*P* = 1.00; Figure 1). Furthermore, in none of these groups did calves shed more than 4 times.

### Association between frequency of shedding and necropsy observations

Overall, the distribution of tissue culture categories and gross lesion scores was not different between shedding and non-shedding calves (*P* = 0.90 and 0.19, respectively; Table 2). However, shedding calves had a higher histology score than non-shedding calves (*P* = 0.08). Conversely, when shedding frequency was taken into account, calves having > 4 fecal culture-positive samplings between inoculation and necropsy had more culture-positive tissue locations and more severe gross and histological lesions compared to less frequent shedders (*P* = 0.03, 0.013 and < 0.001, respectively).

**Table 2:** Fecal culture results compared to tissue culture, histology and macroscopy determined at necropsy

**Fecal culture**^**1**^	**Tissue culture**^**2**^				**Histology**^**3**^				**Macroscopy**^**4**^				
	**0**	**1**	**2**	**3**	**0**	**1**	**2**	**3**	**0**	**1**	**2**	**3**	**4**
0	8^†^	9	1	0	6	11	1	0	10	0	2	8	0
1-4	13	13	1	0	2	24	1	0	9	4	0	12	0
> 4	1	1	1	2*	0	1	2	2*	0	0	0	3	2*

^†^Number of calves assigned to each category; 50 in total. Calves in total were experimentally inoculated with MAP.

^1^Number of fecal culture-positive samplings starting 2 weeks after inoculation until necropsy.

^2^0 = no tissue locations culture-positive; 1 = 1–3 tissue locations culture-positive; 2 = 4–6 tissue locations culture-positive; 3 = > 6 tissues culture-positive.

^3^0 = no lesions; 1 = focal lesions; 2 = multifocal lesions; 3 = diffuse lymphocytic, multibacillary or intermediate lesions.

^4^0 = no macroscopic changes; 1 = one enlarged or edematous lymph node of the small intestine or liver; 2 = multiple enlarged and edematous mesenteric lymph nodes and/or hyperemia of the ileocaecal valve; 3 = enlarged mesenteric lymph node(s) and/or mild to moderate thickening of ileal or jejunal mucosa; 4 = enlarged mesenteric lymph node(s) and severe thickening and corrugation of the ileal, jejunal and colon mucosa.

*These 2 calves had clinical signs of JD and were euthanized at 16 months of age.

### Interval inoculation to shedding

**Figure 3:**
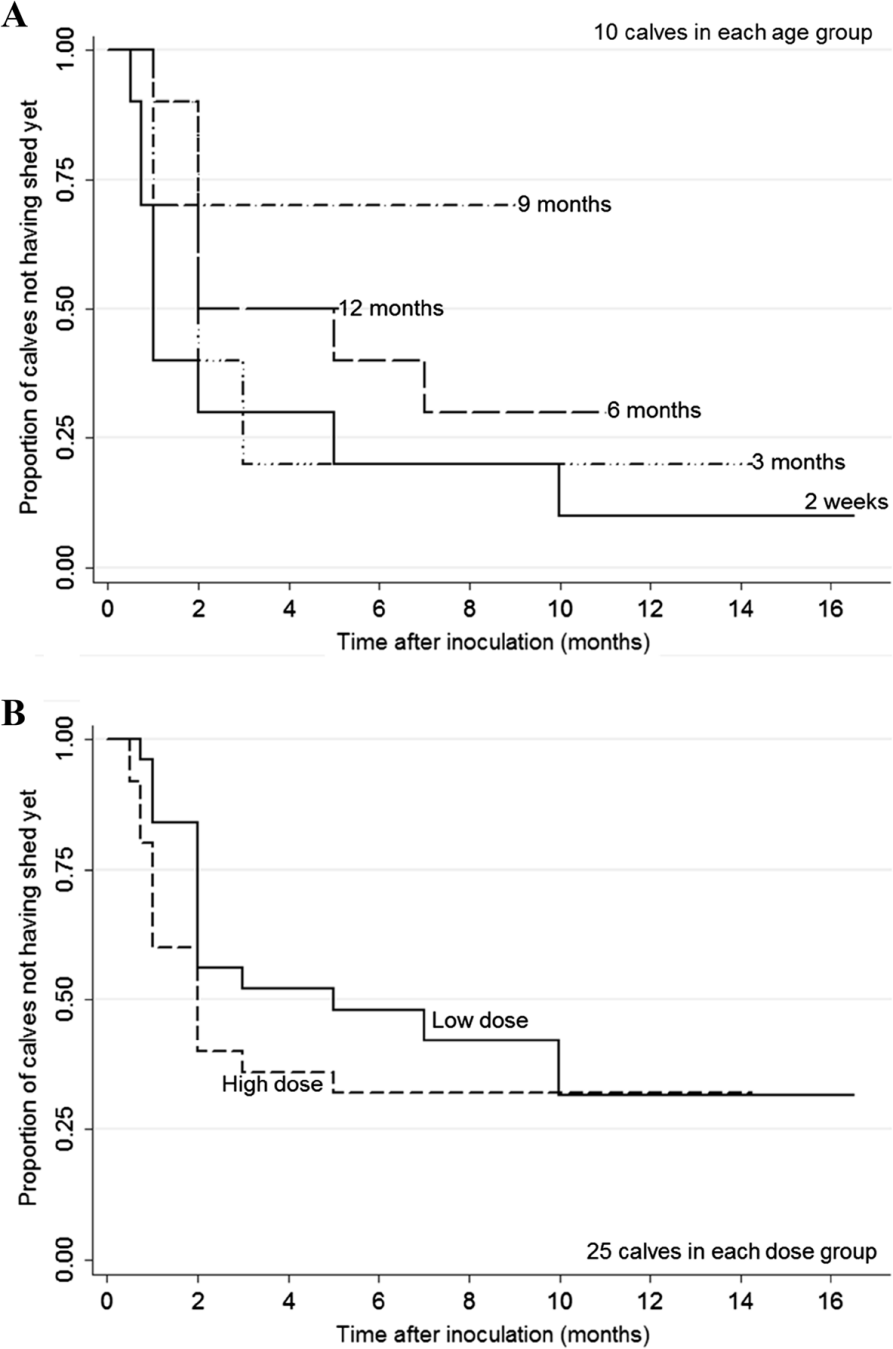
**Kaplan-Meier curve for time to first positive fecal sample. A)** per age group, and **B)** per dose group. Proportion of calves not having shed yet are plotted for each month after inoculation; each curve represents all 10 calves each of the 5 age groups or all 25 calves in each dose group in **A)** and **B)**, respectively.

Interval to first positive fecal culture was not different between the HD and LD calves (*P* = 0.25); however, the interval between inoculation and first positive fecal culture increased with increasing age (*P* = 0.07; Figure 3).

## Discussion

Calves in all 5 age groups and both dose groups shed MAP in their feces, and sporadic, intermittent as well as continuous shedding was detected. Calves inoculated at a young age with a HD shed MAP more frequently than LD calves. However, in older age groups (6, 9 and 12 months), this dose-dependent effect was no longer present. Although shedding usually started within 6 months after inoculation (and peaked at 2 months), calves inoculated at an older age usually started shedding later after inoculation. Finally, frequently shedding calves had more severe gross and histological lesions and more MAP culture-positive tissue locations.

Even though older calves are still susceptible to MAP infection [20], calves inoculated up to 3 months old in particular shed MAP more frequently, especially when inoculated with a HD of MAP. This was consistent with previous reports that when calves are exposed at a young age in particular to a HD of MAP, shedding and clinical signs of JD will develop sooner after infection [19],[26],[27]. However, it is unclear why this dose difference disappeared when calves were inoculated at 6, 9 or 12 months of age. Although infection under field conditions is unknown, infection pressure on a farm regularly having clinical JD cases is likely higher than a farm with only subclinical cases. Moreover, heifers on high-prevalence farms shed more often compared to heifers on low-prevalence farms [26]. Two doses of inoculum were used, a HD of 5 × 10^9^ CFU given on 2 consecutive days (5 times the recommended standard bovine challenge dose [21]) and a LD of 5 × 10^7^ CFU also given on 2 consecutive days (10 times higher than the lowest confirmed and consistent infectious dose for young calves [28]). Even though a controlled infection trial cannot determine the bacterial burden on farm, we inferred that these two doses represented realistic infection pressures under field conditions. Calves inoculated at 2 weeks or 3 months with a HD of MAP shed more frequently, had more severe gross and histology lesions and more culture-positive tissue locations. Therefore, prevention of infection of young calves, in combination with lowering the infection pressure, remains an essential component of JD control programs.

Based on the general assumption that only younger calves are susceptible to MAP infection [27], older calves are rarely included in MAP challenge experiments [18]. However, in this experiment, it was noteworthy that calves inoculated at 6, 9, or 12 months became infected [20], and 40% shed early (at 1 and 2 months after inoculation; Figure 1) after inoculation. Although this apparent lack of dose dependency in the 6-, 9- and 12-month inoculation groups was not expected a priori, susceptibility of calves up to at least 1 year of age, even with a low dose of MAP, should be considered in control programs.

Shedding was most frequent in the first 6 months after inoculation and peaked 2 months after inoculation. A meta-analysis of MAP challenge experiments concluded that the median time to first shedding was 3 months, whereas most shedding was detected within 6 months after inoculation [18]. Other studies documenting early shedding that were not included in that meta-analysis confirmed its findings: shedding peaked at 2–4 months after oral inoculation in 4 calves receiving an oral dose of 10^11^-10^12^ CFU of MAP [29] and in 20 calves inoculated orally with 10^8^ CFU of MAP [30]. The peak in shedding probably depends on the dose of inoculation; in calves inoculated with a HD of MAP, the peak occurred sooner after inoculation [19]. Additionally, in a clinical trial involving 56 calves, this shedding peak was not reduced after Hsp70 vaccination, even though the candidate vaccine reduced shedding after this initial peak [31]. A peak in shedding shortly after inoculation also occurred in 4 white-tailed deer inoculated with an oral dose of 10^10^ CFU of MAP [32] and in a vaccination trial including 16 goats orally inoculated with 10^9^ CFU of MAP [33]. Early shedding was also observed in 38 orally inoculated sheep (dose 10^7^-10^8^ CFU) [34]. Also, in a mouse model, a peak in fecal shedding occurred 4 months after inoculation, whereas only a low bacterial burden was detected in intestinal tissues [35]. At 5 months after inoculation, the number of bacteria in the tissues was still increasing progressively [35]. An early peak in shedding was in this case not consistent with the general assumption that high shedding is associated with a higher bacterial burden in the tissues. Clearly, there is an urgent need to elucidate mechanisms behind translocation of MAP to the intestinal lumen and subsequent shedding in feces. Unfortunately, the underlying cause of fecal shedding is currently unknown [36]. This peak did not coincide with age-related and developmental changes of the calves, for example weaning, as this peak occurred in all 5 inoculation age groups. Therefore, this peak was more likely due to temporal changes in host cells containing MAP. Previously, it was suggested that infected macrophages will emigrate from the mucosa into the intestinal lumen and consequently passed in the feces, consistent with intermittent shedding [37],[38]. Others have theorized that shedding of MAP comes from burst macrophages in the mucosa producing extracellular bacteria, and if these bacteria remain extracellular and translocate to the lumen, this would explain increased shedding of MAP in feces [39]. However, none of these hypotheses explain a peak in shedding within the first 6 months, rather than a progressively increasing level of shedding throughout MAP infection. Understanding this shedding mechanism will likely explain this early peak in shedding.

Frequently shedding calves had more severe gross and histological lesions, and more culture-positive tissue locations. Dissemination of MAP in multiple organs was observed in high shedders [40],[41], corresponding with animals in a clinical stage of infection. Lesions at necropsy were more severe in calves that shed frequently. Even though only 2 of the 5 frequently shedding calves in this study had clinical signs, typically coinciding with consistent shedding and severe necropsy lesions [8], we expected that frequently shedding calves without clinical signs also would have more positive tissue locations and more severe gross and histological lesions. Additionally, it was reported that MAP can disseminate before appearance of clinical signs [20],[41] and cause positive tissue cultures. This could account for frequently shedding calves without clinical signs having more positive tissue locations and more severe gross and histological lesions compared to sporadically shedding calves. Presumably, clinical signs would have subsequently appeared in these frequently shedding calves if the study had not been completed at 17 months of age.

One of 6 non-inoculated calves had 3 positive fecal cultures (at 3.5, 4 and 7 months after inoculation) and was also positive on histology, suggesting a true infection with MAP. Even though all calves in this study were housed individually and strict biosecurity measures were applied to avoid cross-contamination, MAP may have been transferred from shedding calves to this control calf. This control calf was not housed adjacent to one of the calves that were shedding constantly, but calf-to-calf transmission (via objects) could have occurred. Recently, dust has also been suggested as a means of transmission for MAP [42]-[45]. It is unlikely that the hay fed to the calves was contaminated with MAP, because the hay was harvested from fields not grazed by cattle for several years. In utero infection is a possibility [46], despite considerable efforts to use calves with minimal probability of an intrauterine infection. The MAP isolates recovered from the infected calves were found to be the same strain used for the inoculation. Based on whole-genome sequencing of over 100 Canadian MAP isolates, the inoculum strain belongs to a lineage that represents approximately 6% of isolates in Canada differing in 200 single nucleotide polymorphisms (SNPs) from the nearest lineage (Ahlstrom et al., unpublished). In addition, PCR amplification of a genomic region, including one of these lineage-specific SNPs (Ahlstrom et al., unpublished) was done on positive fecal culture samples selected from shedding calves representing all age and dose groups in the experiment. All sequences recovered from the fecal isolates shared the specific inoculum lineage SNP; because only 6% of isolates found in Alberta belong to this lineage, it can be concluded with confidence that the isolates were derived from inoculum strain Cow69. Unfortunately, this method was unsuccessful in amplifying the genomic region around this SNP for the shedding control calf and gaining insight regarding the source of infection was not possible. Additionally, whole-genome sequencing of MAP isolates recovered from the ileum and ileocecal valve of Calf 5 were in full agreement with the inoculum Cow69 genome.

Calves in the carrier/subclinical stage of JD may have shed MAP at low levels [47] and since sensitivity of fecal culture is relatively low (23 - 74%, depending on gold standard and definition used) [48], some shedding might have been missed in this study. Although more frequent sampling (weekly, daily) might have increased the detection rate of MAP in feces, this would have been very costly. Additionally, quantification of MAP was not done because the levels of shedding were expected to be low [47] in most calves; consequently, quantitative techniques (e.g. direct qPCR) would not have provided much additional information.

Even though passive shedding could be mistaken for active shedding due to infection, there was no reason to believe this was the case. In this experiment, passive shedding was detected in the days immediately following oral inoculation of MAP, although calves subsequently tested negative 1 week after inoculation, consistent with previous reports [21],[22]. Therefore, shedding 2 weeks after inoculation was considered active shedding, as suggested [21].

In all MAP challenge experiments of younger calves [18],[19],[31], a high proportion of calves shed the bacteria relatively soon after infection. In this study, shedding was detected over the entire period of 16 months. Due to increased adoption of acidified milk feeding and automatic milk feeders, many dairy calves are group-housed both before and after weaning, with potential for calf-to-calf transmission. Therefore, as a next step, an experiment should be done in which inoculated calves are kept in groups with non-infected calves to confirm whether MAP is transferred between calves. Also, the presence of shedding of young stock on dairy farms should be determined. If these 2 steps confirm calf-to-calf transmission, JD control programs should be adjusted to include prevention of calf-to-calf transmission of MAP.

When minimizing exposure of calves to manure, this will benefit reduction of MAP-infection as well as reduction of other fecal-orally transmitted diseases [2],[7]. Preventive measures for young calves should be extended to calves up to 1 year of age (based on this trial). However, naturally exposed heifers became infected [49], arguably all age references should be removed from control programs, because older calves are still susceptible to MAP infection and shedding as a consequence. Reducing infection pressure in general is important to keep the infection dose low and thus reduce the effects of JD.

To conclude, calves inoculated with MAP up to 1 year of age shed MAP shortly after inoculation, with a peak 2 months after inoculation. Some calves inoculated with a HD shed continuously. This could result in contamination of the environment of calves, and when group-housed lead to calf-to-calf transmission. Prevention programs may be more effective if calves up to 1 year of age are considered both susceptible to MAP infection and a potential source of infection for other calves.

## Abbreviations

BHI: Brain heart infusion

HD: High dose

HPC: Hexadecylpyridinium chloride

JD: Johne’s disease

LD: Low dose

MAP: *Mycobacterium avium* subspecies *paratuberculosis*

## Competing interests

The authors declare that they have no competing interests.

## Authors’ contributions

Designed and conducted the experiment: RM, HWB, KO, JDB. Developed and conducted inoculum preparation and the inoculation procedure, collected and analysed fecal samples, performed necropsies: RM. Animal management, health and welfare: RM, KO, RW, JDB. Analysis of data: RM, HB. Drafted the manuscript: RM, HWB, JDB. All authors read and approved the final manuscript.
